# Supplementary material for: Individualised computational modelling of immune mediated disease onset, flare and clearance in psoriasis
Source: PLoS Comput Biol. 2022 Sep 30;18(9):e1010267. doi: 10.1371/journal.pcbi.1010267 (PMC9524682; doi:10.1371/journal.pcbi.1010267)
Supplement: S1 Text — A. Model details. B. Bimodal behaviour of the model. C. Parameter sensitivity analysis. D. Growth and differentiation rate of keratinocytes. E. Keratinocytes growth vs. epidermis cell density. F. Apoptosis and Desquamation Rates. G. Supplementary figures. Fig A. Phase planes demonstrating the effect of changing initial values of DC and TA species on converging to the two stable steady states of the model. Fig B. Parameter sensitivity analysis. Fig C. Model simulations of 1x weekly UVB phototherapy (30 doses) vs. UVB sensitivity. Fig D. Model simulation of UVB-induced cell apoptosis vs. cell growth arrest. Fig E. Modelling flares by introducing an immune stimulus to the model. (PDF) [file pcbi.1010267.s001.pdf]

Individualised computational modelling of immune mediated disease onset, flare and clearance in psoriasis

Fedor Shmarov[1,2], Graham R. Smith[3], Sophie C. Weatherhead[4], Nick J. Reynolds[5,*], and Paolo Zuliani[1,*]

[1]School of Computing, Newcastle University, UK
[2]Department of Computer Science, University of Manchester, UK
[3]Bioinformatics Support Unit, Newcastle University, UK
[4]Department of Dermatology, Royal Victoria Infirmary, Newcastle Hospitals NHS Foundation Trust, UK
[5]Translational and Clinical Research Institute, Newcastle University, UK

[*]joint senior authors: nick.reynolds@newcastle.ac.uk (NJR); paolo.zuliani@newcastle.ac.uk (PZ)

Supplementary Material

A Model details

We used the SMT solver dReal to show that there are only 3 balls of diameter at most $3 \cdot 10^{-9}$ in the domain $[10^{-9}, 10^{32}] \times \cdots \times [10^{-9}, 10^{32}]$ that can contain the solutions. (Although this does not formally guarantee that there are at least 3 solutions.) So if there are more than one solution within each such ball (there are 32 solutions overall), they are at most $3 \cdot 10^{-9}$ far from each other. Below are the intervals where the solutions for each species might be. So basically there are no solutions in the regions $\pm 10^{-9}$ from the right and the left bounds of the intervals below.

Solution intervals group 1 (healthy state):

$$A \in [7.982009560747935, 7.982009560748187]$$
$$D \in [50529.35801361269, 50529.35801361356]$$
$$DC \in [1563.063946375348, 1563.063946375427]$$
$$GF \in [717.0296163340762, 717.0296163341161]$$
$$IL_{17/22} \in [21.31633768900653, 21.31633768900844]$$
$$IL_{23} \in [42.82366976370817, 42.82366976371033]$$
$$SC \in [3947.21384224557, 3947.213842245908]$$
$$T \in [1556.092651297535, 1556.092651297615]$$
$$TA \in [22224.36715394875, 22224.36715394933]$$
$$TNF \in [21.31633768900732, 21.31633768900842]$$

Solution intervals group 2 (transition state):

$$A \in [9.942521279320136, 9.942521279321062]$$
$$D \in [63458.54551987114, 63458.54551987203]$$
$$DC \in [1905.499517136538, 1905.499517136938]$$
$$GF \in [881.2100494062016, 881.210049406315]$$
$$IL_{17/22} \in [25.98631442285044, 25.9863144228516]$$
$$IL_{23} \in [52.20546622292645, 52.20546622292981]$$
$$SC \in [4855.45800768624, 4855.458007687109]$$
$$T \in [1897.000952867967, 1897.00095286809]$$
$$TA \in [27308.70879564229, 27308.7087956429]$$
$$TNF \in [25.9863144228494, 25.98631442285055]$$

Solution intervals group 3 (psoriatic state):

$$\begin{aligned}
A &\in [26.59853719540063, 26.59853719540152] \\
D &\in [173504.1395710201, 173504.139571021] \\
DC &\in [4804.398597448669, 4804.398597449334] \\
GF &\in [2271.064736922396, 2271.064736922519] \\
IL_{17/22} &\in [65.52014914891787, 65.52014914891896] \\
IL_{23} &\in [131.6273588342101, 131.6273588342123] \\
SC &\in [12544.22751851258, 12544.22751851354] \\
T &\in [4782.970887871005, 4782.970887871085] \\
TA &\in [70349.63537915527, 70349.63537915586] \\
TNF &\in [65.52014914891788, 65.52014914891897]
\end{aligned}$$

B Bimodal behaviour of the model

Our model presented in Fig 1b and equations (1) features bimodal behaviour with two stable positive real (*i.e.,* "healthy" and "psoriatic") steady states and one unstable positive real (*i.e.,* "transition") steady state (see Table 2 for the corresponding cell densities in the three states). The transition from "healthy" to "psoriatic" state happens via an introduction of a sufficiently strong immune stimulus (this causes an immediate increase in the number of dendritic cells), while the transition from "psoriatic" to "healthy" state can be achieved through inducing a sufficient amount of apoptosis in the proliferating keratinocytes and the immune cells. In Fig A in we show phase planes demonstrating the behaviour of the model depending on the initial density of dendritic cells and transit-amplifying cells, which are the two key species involved in the transition between the steady states.

C Parameter sensitivity analysis

To evaluate the robustness of our model with respect to rounding changes in key parameter values, we performed a sensitivity analysis for the model parameters from Table 1 whose values feature more than two significant digits (*i.e.,* sc_2, sc_{2ta}, sc_{20}, ta_2, ta_{20}, ta_{2d}, d_{20} and d_{desq}). We analysed steady states for 256 combinations of these parameters by rounding their values up and down to two significant digits. (Specifically, we used MATLAB to find all the zeroes of the right-hand side of the ODE system (1), initialised with each of the 256 combinations.) As a result, our model features bistability (*i.e.,* presence of two stable steady states; see Fig B) for 200 parameter combinations, while in the remaining 56 cases bistability was broken (*i.e.,* the model featured only one steady state). The results show that our model is generally robust with respect to small variations of the parameter studied. We note that the higher value of d_{desq} is always necessary to break bistability. In addition, the yellow column suggests that d_{desq} and ta_{2d} are the two major 'culprits'.

D Growth and differentiation rate of keratinocytes

The growth rate and the differentiation rate of keratinocytes are the positive terms of the equations (1) for cell species SC, TA, and D. The terms are $(TNF\alpha + IL_{17/22})(sc_2 SC + sc_{2ta} SC + ta_2 TA)$ and

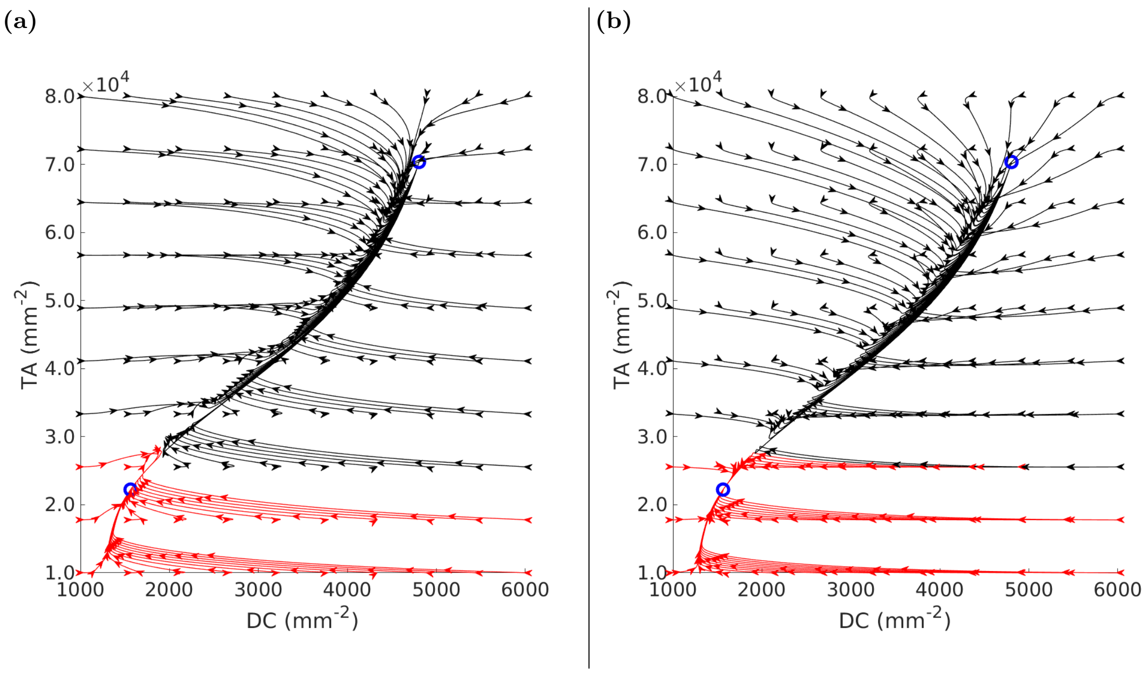

Fig A. Phase planes demonstrating the effect of changing initial values of DC and TA species on converging to the two stable steady states of the model. Red and black arrows represent model trajectories converging to the healthy (lower blue circle) and the psoriatic steady state (upper blue circle), respectively. Panels (a): all the remaining species were initialised to the healthy steady state; (b) remaining species initialised to the psoriatic steady state.

$ta_{2d}(TNF\alpha + IL_{17/22})TA$ for the growth rate and differentiation rate, respectively. Thus, the change of keratinocytes growth rate in the two stable states of the model (healthy and psoriasis) can be calculated as the ratio:

$$\Delta_{KC} = \frac{(TNF\alpha^P + IL^P_{17/22})(sc_2SC^P + sc_{2ta}SC^P + ta_2\,TA^P)}{(TNF\alpha^H + IL^H_{17/22})(sc_2SC^H + sc_{2ta}SC^H + ta_2\,TA^H)}$$

where the superscripts P and H represent the values of the model species in the psoriatic and the healthy steady states, respectively. By utilising the values found in Table 2 we compute $\Delta_{KC} \approx 9.74$.

Similarly, the change in the differentiation rate is $\Delta_D = \frac{ta_{2d}(TNF\alpha^P + IL^P_{17/22})TA^P}{ta_{2d}(TNF\alpha^H + IL^H_{17/22})TA^H} \approx 9.73$.

E Keratinocytes growth *vs.* epidermis cell density

In our model we assume that cell growth rates depend on the cell density. This assumption was made from observing the dependency between epidermal turnover time and epidermal thickness. Turnover time is the amount of time needed to replace all cells in a particular compartment. It can be calculated as the total number of cells in the compartment divided by the rate of cell growth (cell

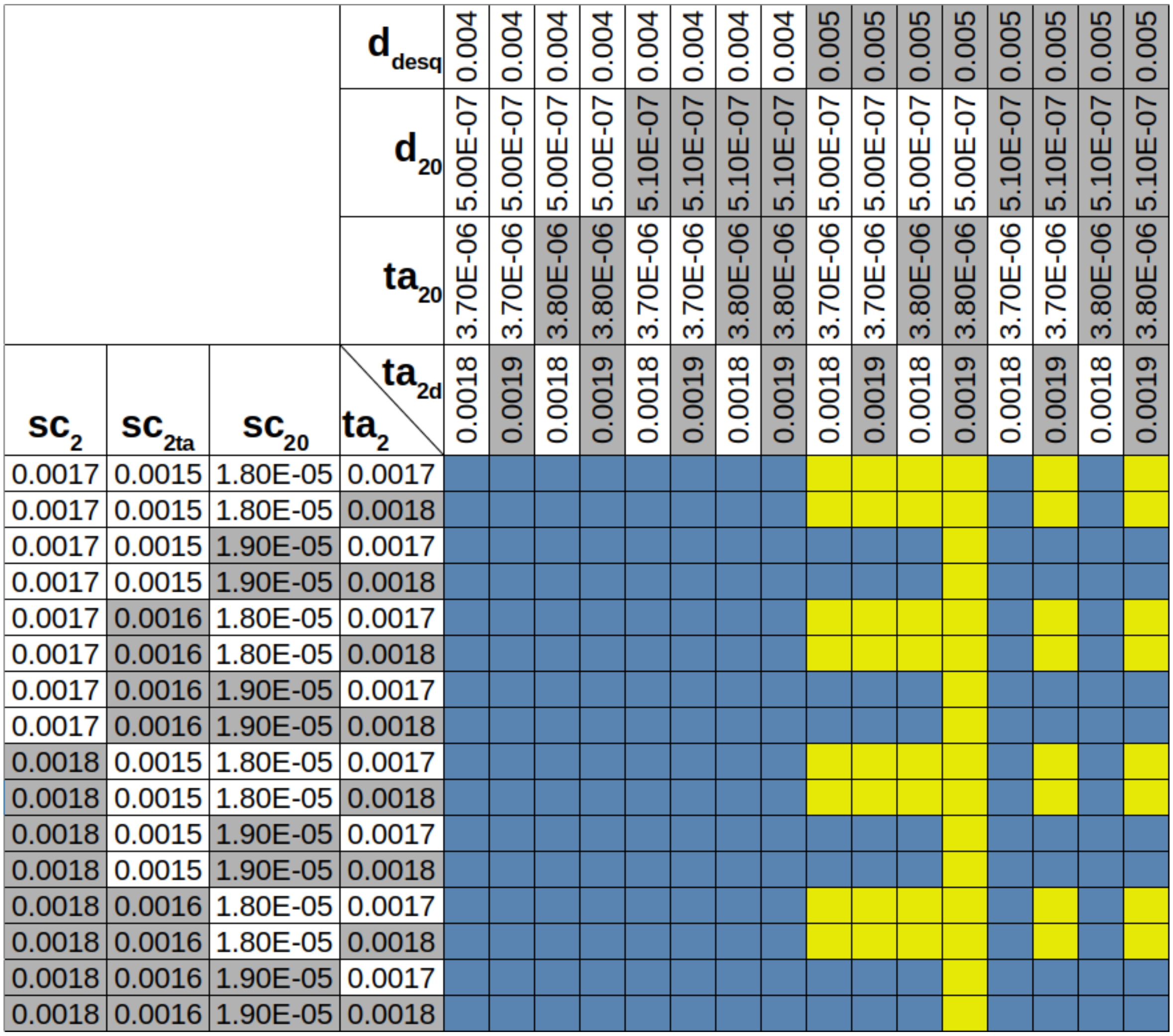

Fig B. Parameter sensitivity analysis. The table shows the effect of changing the model's parameters' values on the number of steady states of the model (cells with grey and white backgrounds contain the upper and the lower parameter values, respectively). Blue cells denote parameter combinations for which bistability holds, while yellow cells denote parameter combinations for which bistability breaks (*i.e.,* the model features only one real positive steady state).

death for systems in homeostasis). It has been reported that the thickness of psoriatic epidermis increases by around 2-5 times [5, SM], while the epidermal turnover time decreases by up to 4 times [5, SM]. (The interested reader may find more references in Section 1.5, 6.3.1 and 6.3.2 of [4].)

Suppose $\tau_H = \frac{\Sigma_H}{\mu_H}$ and $\tau_P = \frac{\Sigma_P}{\mu_P}$ (where Σ_H and Σ_P are total cell densities in healthy and psoriatic epidermis, and μ_H and μ_P are the rates of cell growth in healthy and psoriatic epidermis) are the turnover times for healthy and psoriatic epidermis, respectively. Typically, the growth rate is

described by the law of mass action as $k \cdot \Sigma$, where k is the growth rate constant. Essentially, it states that the cell growth rate depends linearly on the cell density. However, we will show why the law of mass action is not directly applicable in our model without making further assumptions (*e.g.,* growth limiting factors, forces, viscosity, limited nutrition, etc.).

Let $n = \frac{\Sigma_P}{\Sigma_H}$ be the ratio between the thickness of psoriatic and healthy epidermis, and let $m = \frac{\tau_H}{\tau_P}$ be the turnover time ratio between psoriatic and healthy epidermis. From the above it follows that $\mu_P = m \cdot n \cdot \mu_H$, which means that epidermal thickness increases n-fold while the cell growth rate increases by $m \cdot n$. This suggests that the growth rates μ_P and μ_H cannot be modelled by the law of mass action as we derive the following contradiction $\Sigma_P = n \cdot \Sigma_H$ (from the thickness ratio formula) and $\Sigma_P = m \cdot n \cdot \Sigma_H$ (from the growth rates equation).

However, if we assume that the cell growth rate is defined by a nonlinear function $\mu = k \cdot \Sigma^p$, where $p > 1$, we get the following system of equations $\Sigma_P = n \cdot \Sigma_H$ and $\Sigma_P^p = m \cdot n \cdot \Sigma_H^p$. Solving it for p yields $p = (\log_n m) + 1$. Thus, we model keratinocyte growth using the following ODE

$$\frac{dX}{dt} = k_1 \cdot X \cdot IL - k_2 \cdot X^p - k_3 \cdot X, \tag{14}$$

where the term $k_1 \cdot X \cdot IL$ describes the growth rate of keratinocytes as a law of mass action with X being the keratinocyte species and IL being the cytokine species, $k_3 \cdot X$ represents cell degradation also modelled by the law of mass action, and $k_2 \cdot X^p$ describes the limiting factors of keratinocyte growth due to an increase in cell density (this could be caused by nutrients limitation and spatial constraints, for example); we call p the "growth limiting constant". (We note that this approach, also known as *logistic growth* because of the RHS of Eq. (14), has been in use since the 19*th* century for modelling growth dynamics that are subject to constraints, such as for example in ecological modelling with the well-know Lotka-Volterra equations for a predator-prey system.)

We emphasise that Eq. (14) is also used for differentiating keratinocytes (D): Eq. (1) for D features a D^p term. While in the equation for stem and TA cells the corresponding term is needed for bistability, the rationale for D is to enforce a decrease in transit time (turnover time for D species) while maintaining the same cell densities proportions in the psoriatic state. In addition, we assume that $n = m$ (and hence, $p = 2$) in our model for simplicity. However, the epidermal thickness and the cell growth rate can be changed using parameters n and m, which provides modelling flexibility.

F Apoptosis and Desquamation Rates

The dynamics of apoptotic cells (A) is governed by the equation $\frac{dA}{dt} = a_{base}(SC + TA + DC + T + D) - a_{20}A$. It is assumed that in healthy epidermis all cells undergo apoptosis at the same rate (relative to their cell mass). The rate of degradation of apoptotic cells is governed by the term $a_{20} \cdot A$, hence their mean lifetime is $\tau_{apop} = \frac{1}{a_{20}} = 40\ mins$ (in [4, Fig 5-5] a median time of 20 minutes is reported from an *in vitro* study, [1, Fig 1] states 1-3 hours for lymphocytes and up to 48-72 hours for keratinocytes), and [2, Fig 1d] demonstrates an apoptotic cell being destroyed within 20-40 minutes in mice). The apoptosis rate in terms of number of apoptotic cells per 1,000 can be calculated as $\frac{1000 \cdot A}{SC+TA+DC+T+D+A} \approx 0.1$ (the median number of apoptotic cells per 1,000 is reported to be 0.1 in untreated psoriatic skin [5, Fig 1b]). The epidermal desquamation rate in the model can be calculated as $d_{desq} \cdot D$, and in the healthy state it is $d_{desq} \cdot D_H \approx 240.16\ cells/(day \cdot mm^2)$, where D_H is the density of differentiated cells in the healthy steady state. A similar figure for human skin is reported by [3].

G Supplementary figures

Figs Ca, Cb and Cc demonstrate three different simulation scenarios where the same 30 UVB doses are administered once per week, but different UVB sensitivity values are simulated. It can be seen that UVB sensitivities of 0.05 and 0.1 do not induce psoriasis clearance, while a UVB sensitivity of 0.15 (*i.e.,* three times the base value) does clear psoriasis. However, we remark that this is a purely hypothetical scenario as patients with different UVB sensitivities are very unlikely to be assigned the same UVB doses. Most likely a patient with higher UVB sensitivity will receive lower UVB doses.

References

1. A. R. Haake and R. R. Polakowska. Cell death by apoptosis in epidermal biology. *Journal of Investigative Dermatology*, 101(2):107 – 112, 1993.
2. K. R. Mesa, P. Rompolas, G. Zito, P. Myung, T. Y. Sun, S. Brown, D. G. Gonzalez, K. B. Blagoev, A. M. Haberman, and V. Greco. Niche-induced cell death and epithelial phagocytosis regulate hair follicle stem cell pool. *Nature*, 522(7554):94–97, Jun 2015.
3. L. M. Milstone. Epidermal desquamation. *Journal of Dermatological Science*, 36(3):131 – 140, 2004.
4. S. C. Weatherhead. The role of apoptosis in uvb-induced clearance of psoriasis. PhD thesis, Newcastle University; `http://hdl.handle.net/10443/1156`, 2011.
5. S. C. Weatherhead, P. M. Farr, D. Jamieson, J. S. Hallinan, J. J. Lloyd, A. Wipat, and N. J. Reynolds. Keratinocyte apoptosis in epidermal remodeling and clearance of psoriasis induced by UV radiation. *Journal of Investigative Dermatology*, 131(9):1916 – 1926, 2011.

(a)

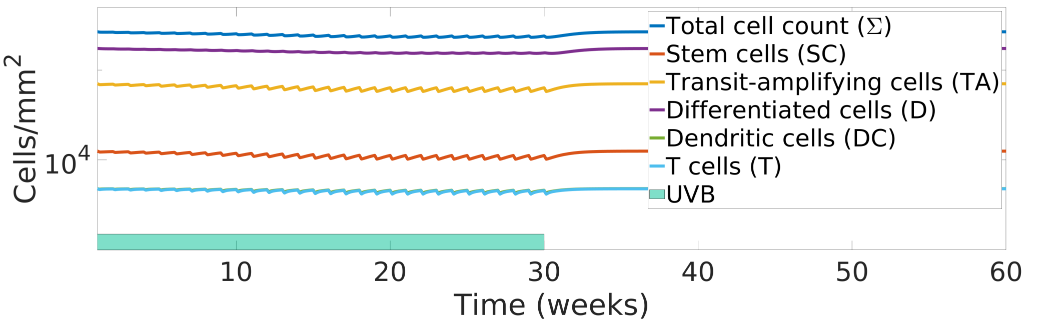

(b)

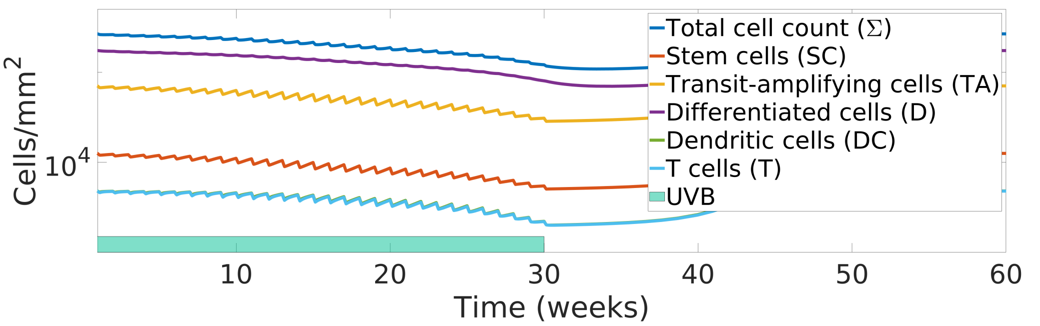

(c)

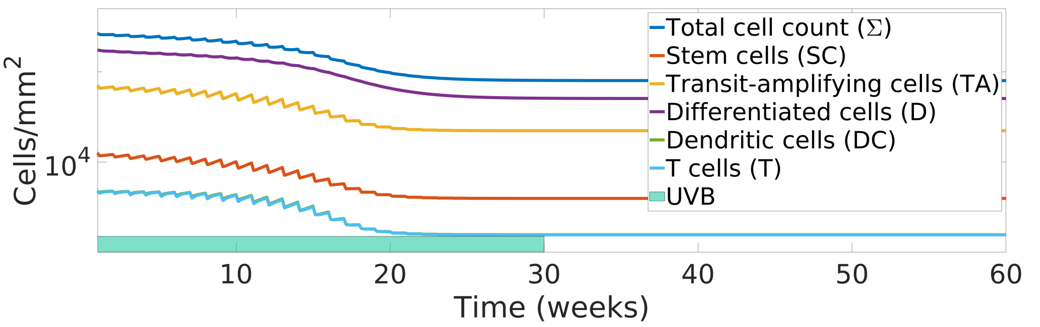

Fig C. Model simulations of 1x weekly UVB phototherapy (30 doses) *vs.* UVB sensitivity. Panels: (a) $uvb_s = 0.05$: no clearance; (b) $uvb_s = 0.1$: no clearance; and (c) $uvb_s = 0.15$: psoriasis cleared.

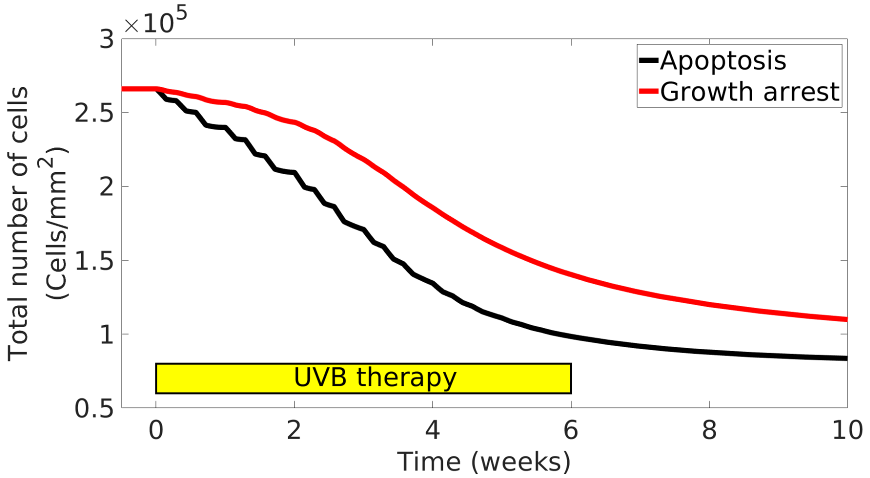

Fig D. Model simulation of UVB-induced cell apoptosis *vs.* cell growth arrest. The Apoptosis model reaches the healthy steady state at the end of the UVB therapy, while the Growth arrest model is still in moderate psoriasis at the end of the therapy and an eventual psoriasis clearance. The latter contradicts clinical observations, and it was thus disregarded.

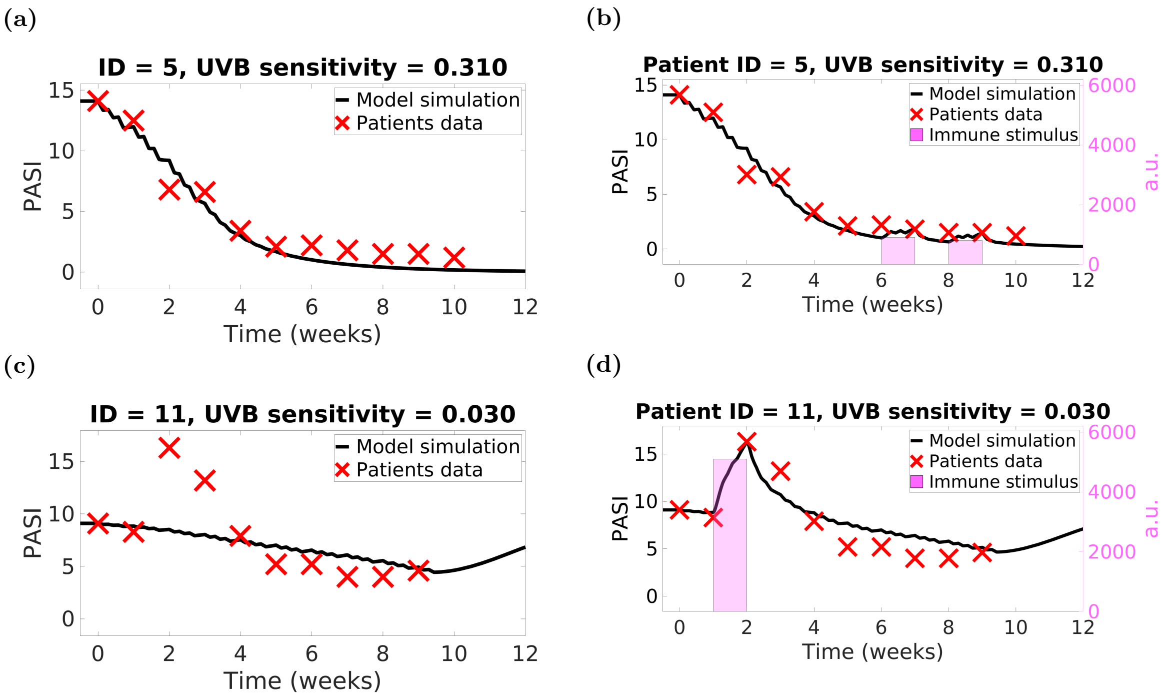

Fig E. Modelling flares by introducing an immune stimulus to the model. Panels (a), (c): two patients PASI trajectories and their model simulation without immune stimulus. Panels (b), (d): the same two patients with their respective model fitted with immune stimuli, allowing a much closer match between patient data and model simulation. Panels (b) and (d) are the same as Figs 6b and 6c, respectively: they are reported here for the reader's convenience.
